# Dietary pattern and other factors of breast cancer among women: a case control study in Northwest Ethiopia

**DOI:** 10.1186/s12885-023-11501-1

**Published:** 2023-11-01

**Authors:** Hiwot Fentie, Peter Austin Morton Ntenda, Fentanesh Nibret Tiruneh

**Affiliations:** 1 Department of Applied Human Nutrition, Faculty of Chemical and Food Engineering, Bahir Dar Institute of Technology, Bahir Dar University, Bahir Dar, Ethiopia; 2 Malaria Alert Centre, Kamuzu University of Health Sciences, Private Bag 360, Chichiri, Blantyre 3, Malawi

**Keywords:** Breast cancer, Women, Dietary patterns, Ethiopia

## Abstract

**Introduction:**

Breast cancer is presently the most commonly diagnosed cancer in women, and it stands as the leading cause of cancer-related deaths worldwide. Notably, breast cancer rates have seen a significant increase in sub-Saharan African countries, including Ethiopia. Several risk factors contribute to breast cancer, some of which can be modified, while others are inherent. Promoting a healthier diet is strongly encouraged as a preventive measure against breast cancer. However, it’s noteworthy that no previous research has investigated the connection between dietary patterns and the risk of breast cancer among Ethiopian women. Therefore, the primary objective of the current study is to examine the relationship between dietary patterns, socioeconomic and behavior factors associated with breast cancer in Ethiopian women.

**Methods:**

A case-control study was conducted at an institution in Bahir Dar, Northwest Ethiopia, involving 260 women, comprising 86 cases and 174 controls. We administered a standardized and validated questionnaire to assess a range of sociodemographic, reproductive, clinical, lifestyle, and dietary characteristics through face-to-face interviews. To analyze the differences between the cases and controls, we employed the Chi-square test. Furthermore, we assessed the relationships between these variables using binary multivariate logistic regression. To measure the association between variables, we utilized odds ratios with 95% confidence intervals.

**Results:**

The results of the multivariate analysis indicated that participants in the younger age group had significantly lower odds of developing breast cancer (AOR = 0.05; 95% CI: 0.00-0.91) compared to those in the older age group. Additionally, women who breastfed their children for shorter durations were 3.66 times more likely to develop breast cancer (AOR = 3.66; 95% CI: 2.78–6.89) than those who breastfed for longer periods. Furthermore, women with sedentary lifestyles faced a significantly higher risk of breast cancer, with odds 10.53 times greater (AOR = 10.53; 95% CI: 5.21–21.36) than their counterparts who engaged in moderate or highly active lifestyles. Lastly, participants who had previously undergone chest therapy were 6.43 times more likely to develop breast cancer (AOR = 6.43; 95% CI: 3.20–13.90) compared to those who had not.

**Conclusions:**

Breast cancer prevention interventions, including breastfeeding counseling and increased physical activity should be recognized as a central strategy for lowering breast cancer risk. Furthermore, healthcare providers should aim to minimize exposure to chest radiation therapy.

**Supplementary Information:**

The online version contains supplementary material available at 10.1186/s12885-023-11501-1.

## Background

Breast cancer is currently the most commonly diagnosed cancer among women and is the leading cause of cancer-related deaths worldwide [1]. Shockingly, one in every eight women globally will face a breast cancer diagnosis during her lifetime [2]. In sub-Saharan African countries, including Ethiopia, the incidence of breast cancer has been on the rise, resulting in high mortality-to-incidence ratios due to advanced stage at presentation and limited access to effective treatments [3]. In Ethiopia, the incidence of breast cancer has shown a continuous and rapid increase year by year. It now stands as the most prevalent cancer, accounting for 33% of all cancers in women and 23% of all cancers overall [4]. What’s particularly concerning is that breast cancer tends to occur at a younger age in Ethiopian women compared to their Western counterparts, often presenting at an advanced stage [5].

While several well-established risk factors for breast cancer, such as family history, early menarche, late menopause, adult-attained height, and other reproductive histories, are challenging to modify [6], diet stands out as a modifiable factor. Extensive research has examined the associations between various single foods and nutrients with breast cancer, revealing that dietary modifications could prevent approximately one-third of breast cancer cases [7]. Presently, the emphasis is on shifting toward a healthier diet, one that favors nutrient-rich and less energy-dense foods. This dietary approach is strongly encouraged by the World Cancer Research Fund (WCRF) as a means to prevent various cancers, including breast cancer [8]. High energy intake and low physical activity significantly contribute to the risk of breast cancer, and adopting healthier dietary patterns is the most impactful way to mitigate this risk [9]. Among specific dietary factors, alcohol consumption stands out as a well-established risk factor for breast cancer [10]. Likewise, high consumption of red meat, animal fats, and refined carbohydrates has been linked to an increased risk of breast cancer [11]. Conversely, compelling evidence indicates that adopting healthy dietary patterns, characterized by increased consumption of fruits, vegetables, whole grains, and dietary fiber, is associated with a reduced risk of breast cancer [12] [13] [14].

While specific foods have been implicated in breast cancer risk in some instances, it’s important to recognize that food is a complex interplay of various nutrients. As a result, the overall body of evidence remains inconclusive [15]. Many epidemiological studies examining the role of dietary patterns in breast cancer have predominantly taken place in developed countries. Dietary patterns are known to vary significantly across populations, influenced by factors such as geography, socioeconomic status, and cultural food habits, preferences, and availability [16]. In the Ethiopian context, the dietary pattern exhibits unique characteristics, primarily consisting of cereals, roots, tubers, and pulses. This diet is characterized by low dietary diversity and limited consumption of fruits, vegetables, fish, and animal products [17].

Due to limited treatment options with poor prognoses, a significant number of Ethiopian women are diagnosed with advanced breast cancer [3]. Consequently, primary prevention strategies, including the promotion of healthy dietary patterns, emerge as the most cost-effective means of reducing cancer incidence rates and high mortality in low-income countries like Ethiopia, where incidence rates are on the rise. To the best of our knowledge, no previous studies have explored the association between dietary patterns and breast cancer risk among Ethiopian women. Therefore, the primary aim of this study is to investigate the relationship between dietary patterns, socio-economic, reproductive and behavioral factors and breast cancer risk among Ethiopian women while controlling for other important covariates. We hypothesized that regular consumption of healthy dietary pattern, high socio-economic status, long duration of breastfeeding and increased physical activity reduce the risk of breast cancer.

## Methods

### Study design and setting

We conducted an institution-based case-control study from May 16 to July 16, 2021, at Felege Hiwot Comprehensive Specialized Hospital (FHCSH) in Bahir Dar, Northwest Ethiopia. In the first phase, we selected all 88 cases of women aged 18 and above with pathologically confirmed breast cancer who were referred to the chemotherapy clinics of FHCSH, which serves as a cancer diagnosis referral center. In the second phase, we chose 175 control participants from among women aged 18 and above admitted to other sections of the same hospital for various non-neoplastic diseases unrelated to long-term modification of diet.

### Sample size determination

The sample size was determined based on several factors: a 1 to 2 ratio of cases to controls, a confidence level of 95%, a power of 80%, and an odds ratio of 2.7. The odds ratio was derived from a prior case-control study conducted in Addis Ababa, Ethiopia. To arrive at the total sample size, we started with 239 individuals. To account for potential non-responses, we added a 10% contingency, resulting in an additional 24 individuals. Therefore, the final sample size was 263 participants, comprising 88 cases and 175 controls.

### Data collection

#### Dietary assessment

The study’s data collection was conducted by trained nurse professionals who conducted private face-to-face interviews and reviewed medical records. These interviewers received training from nutritionist professionals. Dietary patterns were categorized into ‘healthy’ and ‘unhealthy’. A healthy dietary pattern includes the daily consumption of fruits, vegetables, and whole grains, as well as never to little (never to 1–3 per month) consumption of processed food, red meat, and sweetened beverages ,.An unhealthy dietary pattern includes 1–2 times per week to daily consumption of fatty and greasy foods, sweetened beverages and foods, highly flavored food, overly pungent food, canned and processed foods, and excessive alcohol consumption includes (1–2 times per week to daily).

Data were gathered using a validated semi-quantitative food frequency questionnaire (FFQ) [18] [19], which was adapted to include Ethiopian food items. This FFQ has previously demonstrated relative validity and reproducibility in assessing food and nutrient intakes among adults. Compared to other dietary assessment methods like short-term recall or diet records, FFQ is more user-friendly, places fewer burdens on respondents, and provides a quick estimate. This makes the FFQ a practical and suitable tool for measuring long-term dietary intake in most epidemiological studies [20], making it an appropriate choice for our study population. Dietary habits for cases were assessed for the year prior to diagnosis, while for controls, it was the year before the interview. Using this FFQ, the consumption frequency of each food item was evaluated on a daily, weekly, or monthly basis. The questionnaire was initially prepared in English, then translated into Amharic, the local language. To ensure consistency, it was translated back into English by a professional translator.

#### Assessment of Breast cancer

Pathologically confirmed breast cancer was the outcome of this study. Pathologically confirmed breast cancer refered to a diagnosis of breast cancer that has been definitively confirmed through the examination of tissue samples (biopsy) obtained from the breast.

#### Assessment of potential sociodemographic factors

We gathered self-reported demographic and socioeconomic information including age (18–29, 30–49 and > 50 years old); marital status (Married/unmarried); residence (urban/rural); religion (orthodox/others); education (illiterate, primary, secondary and college and above); occupation (house wife, self-employed, government employed); and income/monthly (≤ 1000, 1001–3000, 3001–6000 and > 6000 birr) was collected. Detailed information was collected regarding history of health, family history of breast cancer, reproductive risk factors, age at menarche (≥ 15/>15 years old) and menopause status (pre-menopause/post-menopause), history of child birth(yes/no), age at first birth (14–18, 19–24 and > 25), breast-feeding history at for each live birth (≤ 1 year/>1 year), and use of modern contraceptives (yes/no). Anthropometric measurements (weight, height) were also collected. BMI was calculated using measured height and weight (kg/m2) [21] [22]. BMI was categorized as under- underweight (18. >18.5 kg/m^2^), Normal weight (18.5–24.9 kg/m^2^) and overweight/obese ( > = 25.0 kg/m^2^). Physical activity level was assessed as sedentary (such as reading, working on a computer, watching television), light activity (including slow waking, standing with minor efforts), moderate activity (such as brisk waking, swimming), very active and, extremely active (including running and carrying heavy load). Cigarette Smoking (yes/no) and alcohol drinking (Never, 1–3/month, 1–2/week, 3–4/week and daily) behaviors were also collected furthermore. We also collected data on breast cancer cases including stage of breast cancer (I-IV) and affected breast (right/left) Previous chest radiation therapy (yes/no) was collected for both case and control patients as well. See the supplementary information (questionnaire) on annex 1.

### Statistical analysis

We conducted data analysis using SPSS version 20, a statistical software package. To evaluate the differences between cases and controls, we employed the Chi-square test, presenting the results as frequencies and percentages. For assessing the relationships between the explanatory variables and breast cancer, we utilized both bivariate and multivariate logistic regression, calculating odds ratios and 95% confidence intervals (95% CI). Variables with a p-value of less than 0.25 in the bivariate analysis were included in the multivariable model. We assessed the goodness-of-fit of each model using the Hosmer and Lemeshow Test, where a higher p-value indicates a better model fit. Importantly, all models demonstrated p-values greater than 0.05.

### Ethical consideration

Since the Bahir Dar Institute of Technology (BiT) lacks an institutional review board, we obtained ethical clearance through our partner institute, the Amhara Public Health Institute (APHI). Furthermore, we ensured the informed consent of all study participants by explaining the study’s objectives. For participants who were unable to read the consent form, healthcare providers read it aloud to them. Those who willingly volunteered to participate provided their consent by either signing the form or applying a thumb impression. We assured respondents that their names would not be disclosed, and all information provided would remain confidential. Additionally, participants were given the opportunity to ask questions about the study and had the option to refuse or discontinue their participation at any time. The APHI research review committee approved that all methods adhered to the applicable ethical standards and regulations outlined in the Declaration of Helsinki.

## Results

Table 1 displays the distribution of selected characteristics among breast cancer cases and control participants. Age showed a significant difference between the two groups, with a higher percentage of younger individuals among the controls (p = 0.000). Additionally, education, occupation, and income status were significantly different between cases and controls, with controls generally having higher levels of education, occupation, and income. Regarding disease and behavioral characteristics, a larger percentage of breast cancer cases had undergone chest radiation therapy (p = 0.000), reported sedentary behavior (p = 0.000), and experienced under-nutrition (p = 0.000). In terms of reproductive-related factors, the duration of breastfeeding significantly varied between cases and controls (p = 0.000). The higher proportion of controls were breastfed their children for longer duration than their case counterparts. All participants belonged to the same ethnic group (Amhara).

**Table 1:** Characteristic of participants among cases and controls in FHCSH, Northwest Ethiopia 2022 (n = 260)

	Category	Control (%)	Case (%)	Total	Mean/SD	X^2^(p-value)
	**Socio-demographic Variables**					
Residence	Urban	103 (70.1)	44 (29.9)	147 (56.5)		0.22
	Rural	71 (62.8)	42 (37.8)	113 (43.5)		
Age	18–29 years	73 (89.0)	9 (11.0)	82 (31.5)	37.9/12.5	0.0001
	30–49 years	72 (57.6)	53 (42.4)	125 (48.1)		
	> 50 years	29 (54.7)	24 (45.3)	53 (20.4)		
Marital status	Married	122 (69.7)	53 (30.3)	175 (67.3)		0.270
	Unmarried	52 (61.2)	33 (38.8)	85 (32.7)		
Educational status	Illiterate	52 (52.0)	48 (48.0)	100 (38.4)		0.000
	Primary	35 (66.0)	18 (34.0)	53 (20.4)		
	Secondary	20 (64.5)	11 (35.5)	31 (11.9)		
	College and above	67 (88.2)	9 (11.8)	76 (29.2)		
Occupation	House wife	43 (56.4)	42 (49.4)	85 (32.7)		0.000
	Self employed	66 (71.0)	27 (29.0)	93 (35.7)		
	Government	65 (79.3)	17 (20.7)	82 (31.5)		
Religion	Orthodox	164 (65.9)	85 (34.1)	249 (95.7)		0.557
	Others	10 (90.9)	1 (9.1)	11 (4.2)		
Monthly income	≤ 1000	10 (40.0)	15 (60.0)	25 (9.6)		0.010
	1001–3000	54 (65.1)	29 (34.9)	83 (31.9)		
	3001–6000	62 (68.1)	29 (31.9)	91 (35.0)		
	> 6000	48 (78.7)	13 (21.3)	61 (23.5)		
	**Behavioral and disease related characteristics**					
Disease stage						
	I		7(8.1%)			
	II		27(31.4%)			
	III		24(27.9%)			
	IV		28(32.6%)			
Affected breast						
	Right		32(37.2%)			
	Left		54(62.8%)			
Chest radiation therapy	Yes	11 (30.6)	25 (69.4)	36 (13.8)		0.001
	No	163 (72.8)	61 (27.2)	224 (86.1)		
Physical activity	Sedentary	5 (16.7)	25 (83.3)	30 (11.5)		0.000
	Light activity	109 (71.2)	44 (28.8)	153 (58.8)		
	Moderate and above activity	60 (77.9)	17 (22.1)	77 (29.6)		
Dietary pattern	Healthy	99 (64.3)	55 (35.7)	154 (59.2)		0.274
	Unhealthy	75 (70.8)	31 (29.2)	106(40.8)		
Nutritional status(BMI)	> 18.5kg/m^2^	10 (30.3)	23 (69.7)	43 (16.5)	21.8/2.6	0.000
	18.5–24.9 kg/m^2^	146 (69.9)	63 (30.1)	209 (80.4)		
	>=25.0 kg/m^2^	18 (100.0)	0 (0%)	18 (6.9)		
Smoking habit	Yes	0	0			
	No	174 (66.9)	86 (33.1)	260(100)		
Alcohol consumption	Never	81 (92.0)	7 (8.0)	88(33.8)		0.000
	1–3 times/month	68 (80.9)	16 (19.1)	84(32.3)		
	1–2 times/week 3-4times/ week	23 (59.0) 2 (10.0)	16 (41.0) 18 (90.0)	39 (15.0) 20 (7.8)		
	Daily	0 (0)	29 (100)	29(11.2)		
	**Reproductive health related factors**					
Modern contraceptive	Yes	129 (68.3)	60 (31.7)	189 (72.7)		0.457
	No	45 (63.4)	26 (36.6)	71 (27.3)		
History of child birth	Yes	134 (64.4)	74 (35.6)	208 (80.0)		0.087
	No	40 (76.9)	12 (23.1)	52 (20.0)		
Age of menarche	≤ 15	117 (70.1)	50 (29.9)	167 (64.2)		0.152
	> 15	57 (61.3)	36 (38.7)	93 (35.8)		
Age at first child birth	14–18	33 (54.7)	29 (45.3)	62 (23.8)		0.087
	19–24	78 (67.8)	37 (32.2)	115 (44.2)		
	≥ 25	22 (75.9)	7 (24.1)	29 (11.2)		
Did you breastfeed any of your children? (n = 208)	Yes	133 (65.5)	70 (34.5)	203 (97.6)		0.102
	No	1 (25.0)	3 (75.0)	4 (1.9)		
How long breastfeed your children?	≤ 1year	15 (27.3)	40 (72.7)	55 (21.2)		0.000
	> 1 year	120 (78.4)	33 (63.6)	155 (58.8)		
How many children do you have?	0	40 (76.9)	12 (23.1)	52 (20.0)		0.398
	1–3	75 (60.0)	44 (37)	119 (45.8)		
	≥ 4	59 (66.3)	30 (33.7)	89 (34.2 )		
Menopause status	pre-menopause	127 (70.6)	53 (29.4)	180 (69.2)		0.064
	post-menopause	47 (58.8)	33 (41.3)	80 (30.7)		

Table 2 presents factors associated with breast cancer risk. The crude analysis (bivariate analysis) indicated that several factors were statistically significant in relation to breast cancer risk, including age, residence, education level, occupation, monthly income, number of children ever born, age at first birth, duration of breastfeeding, physical exercise status, age at menarche, and menopause status. However, there was no observed association between dietary patterns and breast cancer risk (OR = 0.74; 95% CI: 0.43–1.27).

**Table 2:** Bivariate and multivariate analysis of factors associated with the risk of breast cancer

Variables	Category	COR with 95% CI	AOR with 95% CI
Age	18–29 years	0.15 (0.60 − 0.35)**	0.05 (0.00-0.91)*
	30–49 years	0.89 (0.47–1.70)	2.09 (0.36–9.30)
	> 50 years	1	1
Residence	Urban	0.72 (0.43–1.21)*	1.33 (0.32–5.45)
	Rural	1	1
Marital status	Married	0.65 (0.40–1.18)	
	Unmarried	1	
Educational status	Illiterate	6.87 (3.10-15.27)**	1.10 (0.78–5.69)
	Primary	3.83 (1.56–9.40) **	8.58 (0.63–11.71)
	Secondary	4.10 (1.49–11.27)**	5.50 (0.39–7.78)
	College & above	1	1
Occupation	Housewife	3.73 (1.79–7.40)**	0.55 (0.06–5.27)
	Self employed	1.56 (0.78–3.14)*	0.65 (0.09–4.97)
	Government emp.	1	1
Monthly income	≤ 1000	5.54 (2.02–151.7)**	2.10 (0.24–8.35)
	1001–3000	1.98 (0.92–4.24)*	0.74 (0.12–4.70)
	3001–6000	1.73 (0.81–3.67)*	0.99 (0.22–4.49)
	> 6000	1	1
Dietary patterns	Healthy	0.74 (0.43–1.27)	
	Unhealthy	1	
Number of children ever born	0	4.6 (0.27–8.28	3.86 (0.33–9.88)
	1–3	1.15 (0.64–1.2.05)	1.04 (0.64–5.69)
	4 and above	1	1
Age at first Birth (n = 208)	14–18	2.60 (0.97–6.95)*	3.86 (0.33–4.48)
	19–24	1.50 (0.58–3.80)	5.70 (0.64–5.69)
	25 and above	1	1
Duration of breastfeeding (n = 208)	<=1year	9.16 (3.25–12.81)**	4.33 (2.78–6.89)*
	> 1 year	1	1
Physical exercise status	Sedentary	17.65 (5.87–3.06)**	10.53 (5.21–1.36)*
	lightly active	1.42 (0.75–2.71)	6.13 (1.042-6.00)*
	Moderate and highly active	1	1
Menopause status	pre-menopause	0.59 (0.34–1.02)*	0.53 (0.10-2.738)
	post-menopause	1	1
Chest radiation therapy	Yes	6.07 (2.81–13.08)**	6.43 (3.20–13.90)*
	No	1	1
Age of menarche	≤ 15years	3.68 (0.40–4.15)*	2.00 (0.76–5.32)
	> 15 years	1	1
Use modern of contraceptive	Yes	0.80 (0.45–1.42)	
	No	1	

Note: **: p < 0.05, *: p < 0.25 for bivariate analysis

*Boldface indicates statistically significant (P < 0.05) for multivariable analysis

The results of the multivariate analysis are shown in Table 2 as well. The age group 18–29 years has a significantly lower risk of breast cancer (AOR: 0.05, 95% CI: 0.003–0.91) compared to the reference group (age > 50 years). Breastfeeding for equal or less than 1 year was associated with a significantly higher risk of breast cancer (AOR: 4.33, 95% CI: 2.78–6.89) compared to breastfeeding for more than 1 year. Additionally, women with sedentary behavior and light physical exercise had 10.53- and 6.13-times higher odds of developing breast cancer (AOR = 10.53; 95% CI: 5.21–21.36 and AOR = 6.13; 95% CI: 1.042-16.00, respectively) compared to their moderately or highly active counterparts. Finally, having received chest radiation therapy was associated with a significantly higher risk of breast cancer (AOR: 6.43, 95% CI: 3.20–13.90) compared to not having received chest radiation therapy.

## Discussion

Our study makes a significant contribution to our understanding of the factors associated with breast cancer risk among Ethiopian women, especially in a region where the prevalence and incidence of this cancer are notably high. The current study found that women’s age, duration of breastfeeding, physical exercise status, and chest radiation therapy were statistically significant associated with breast cancer. Despite the fact that this study has significant limitations due to its reliance on self-reported data and recall bias, it is the first to investigate the connection of dietary patterns and other factors with breast cancer risk in Ethiopia.

Contrary to our initial hypothesis and in contrast to the findings of previous studies [23] [24] [25], our research did not identify any association between dietary patterns and the risk of breast cancer. This unexpected outcome could potentially be attributed to methodological considerations. Food frequency questionnaires, as a retrospective method, involve querying participants about their food and drink consumption patterns over extended periods. This approach relies on participants’ memory, literacy, and numerical skills, which can introduce inaccuracies and subjectivity into the reported data. Responses to dietary questions can result in both over-reporting and under-reporting.

This study uncovered a notable finding: younger participants demonstrated a lower likelihood of developing breast cancer compared to their older counterparts. This observation aligns with extensive evidence indicating an increased breast cancer risk with advancing age [26] [27] [28]. Several factors might contribute to this lower incidence of breast cancer in younger women.

Firstly, hormonal differences [29] could play a role, as well as varying exposure to risk factors like alcohol consumption [29] [30] and obesity [31], which tend to be more prevalent among older women. Additionally, research has consistently shown that younger women are more inclined to engage in regular exercise when compared to their older counterparts [32]. Regular physical activity carries various health benefits, including a potential reduction in the risk of breast cancer [32]. Similarly, overweight and obesity rates tend to be higher among older women [33] [34]. Several studies have highlighted obesity as a significant risk factor for various cancers, including breast cancer [35] [36].

In line with findings from studies conducted in other countries [26] [37] [38] [39], our research uncovered a significant link between breastfeeding duration and breast cancer risk. Specifically, our study revealed that women who breastfed their children for a year or less faced a 3.66 times higher risk of developing breast cancer compared to those who breastfed for over a year. This association can be attributed to the mechanisms by which breastfeeding reduces the risk of breast cancer. Notably, breastfeeding has been shown to lower the levels of hormones such as estrogen and progesterone, which have been linked to an increased risk of breast cancer. Additionally, breastfeeding leads to a shorter menstrual cycle in lactating mothers, limiting the exposure of breast tissue to these hormones and thereby inhibiting the development of cancer cells. Furthermore, breastfeeding promotes the shedding of breast tissue, aiding in the removal of cells with potential DNA damage within the breasts [40]. As such, breastfeeding has emerged as an exceptionally effective method for protecting mothers from breast cancer. In light of these findings, breast cancer interventions, such as breastfeeding counseling and programs aimed at enhancing breastfeeding practices, assume critical importance in reducing the risk of breast cancer.

This study uncovered a statistically significant relationship between physical activity levels and the risk of breast cancer. Notably, women who engaged in sedentary behavior faced a higher risk of developing breast cancer compared to those who maintained moderate or vigorous physical activity. This finding is consistent with previous research, which has consistently shown that regular exercise can significantly lower the risk of breast cancer [41] [42] [43]. The mechanisms through which exercise helps to reduce breast cancer risk are multifaceted. Firstly, exercise can contribute to a reduction in body fat [44], which, in turn, lowers the levels of certain hormones, such as estrogen, known to promote the growth of breast cancer cells. Additionally, physical activity can strengthen the immune system and reduce inflammation [32] [45], both of which are factors that may play a role in the initiation and progression of cancer. Given these compelling findings, it is imperative to view increasing physical activity as a central strategy in the effort to lower the risk of breast cancer.

Consistent with findings from other studies [46] [47], our research revealed that participants who had undergone chest radiation therapy were at a higher risk of developing breast cancer compared to their counterparts. Notably, a history of radiation exposure from chest X-rays was associated with an elevated risk of breast cancer. The underlying mechanism behind this association lies in the direct impact of radiation on the structure of the DNA double helix. This radiation-induced damage triggers DNA damage sensors, leading to processes such as apoptosis and necrosis, disrupting normal mitotic events, and ultimately reshaping various biological characteristics of neoplasm cells [47].

It is important to acknowledge potential limitations when interpreting these results. One notable limitation is the possibility of misclassification due to our reliance on self-reported dietary patterns. Additionally, the lengthy recall periods could lead to inaccurate reporting of past exposures. In light of these limitations, future studies should aim to address these issues for a more comprehensive understanding of the topic.

## Conclusion

In our study, we identified several statistically significant associations with breast cancer risk, including women’s advanced age, duration of breastfeeding, physical exercise status, and chest radiation therapy. These findings underscore the importance of focusing on primary prevention strategies. Interventions should prioritize counseling on optimal breastfeeding duration, encouraging moderate to vigorous physical activity, and emphasizing the need for healthcare providers to limit chest radiation therapy exposure. These measures collectively contribute to reducing the risk of breast cancer.

### Electronic supplementary material

Below is the link to the electronic supplementary material.

Supplementary Material 1

## Data Availability

The datasets analyzed during the current study will be available from the corresponding author on reasonable request.

## List of Abbreviations

AOR: Adjusted Odds Ratio
BMI: Body mass index
CI: Confidence Interval
COR: Crude Odds Ratio
FFQ: Food Frequency Questionnaire
FHCSH: Felege Hiwot Comprehensive Specialized Hospital
