# Supplementary material for: Dietary pattern and other factors of breast cancer among women: a case control study in Northwest Ethiopia
Source: BMC Cancer. 2023 Nov 1;23:1050. doi: 10.1186/s12885-023-11501-1 (PMC10619250; doi:10.1186/s12885-023-11501-1)
Supplement: Supplementary file 1 — Supplementary Material 1 [file 12885_2023_11501_MOESM1_ESM.docx]

**Annex**

**INFORMATION AND CONSENT SHEET (ENGLISH VERSION)**

Hello, my name is ----------. I am working with __________who come from Bahir Dar University, Institute of technology for conducting research entitled: Dietary Pattern and Risk of Breast Cancer among Women: A Case Control Study

I will ask you some questions about your general health and will measure your height and weight. The interview will take just 30 minutes. You will not get any harm by participating in this interview. Your participation will have great role for success of this work and for future researches and programs. Your participation in this interview is voluntarily. You have full right to withdraw the interview any time you get bored. The information we collect from you will be entirely be confidential. The data I collect will be used only for purpose of this research and your name and personality not be identified in the report. Finally, I would like to assure that there is no any risk that would be happens by involving in the study.

For detail information you can contact the investigator through cell phone: +251934592925 and e-mail hiwotfentie1@gmail.com*.*

If you have any questions about the survey, I am ready to respond. Do you have any questions? Please let me know if anything I have stated is not clear and I will be happy to explain it further to ensure you understand.

I have been informed about the purpose and use of this particular research. After all these I understood and:

1. I agree to participate in this research voluntarily

2. I didn’t agree to participate in this research

Date _________

***Thank you in advance for your cooperation to the study!***

**QUESTIONNAIRE (ENGLISH VERSION)**

Card no. __________

Interviewer name __________________________ Date ____________

**I. Personal, socio demographic, anthropometric and clinical information**

| **No** | **Questions** | **Coding categories** | **Remark** |
| --- | --- | --- | --- |
| 101. | Age | _______________ |  |
| 102. | Height | ______________meters |  |
| 103. | Weight | _______________kg |  |
| 104. | What is your religion | 1. Orthodox 4. Adventist 2. Muslim 5. Catholic 3. Protestant 6. Other_________ |  |
| 105. | What is your current marital status | 1. Single 3. Divorced 2. Married 4.Widowed |  |
| 106. | Place of residence? | 1. Urban 2. Rural 3. Semi- urban |  |
| 107. | What is your education level | 1. No education 2. Read and write 3. Elementary (1-4) 4. Primary (5-8) 5. Secondary (9-12) 6. Collage and above 7. Others (specify) |  |
| 108. | What is your occupation | 1. No work/ House wife  2. Daily laborer  3. Farmer  4. Merchant  5. Private Business  6. Government employee  7. Other(specify)___________ |  |
| 109. | What is your estimated family monthly income? | ______________________ |  |
| 110. | Have you ever smoke cigarette? | 1. Yes 2. No |  |
| 111. | Did any of your parents, siblings, or children have breast cancer? | 1. Yes 2. No 3. Don’t know | If NO/Don’t know, go to Q- 113 |
| 112. | If yes, what is your relationship with her? | _______________________ |  |
| 113. | Have you ever had radiation therapy to the chest or breast? | 1. Yes  2. No |  |
| 114. | How do you rate your physical activity level? | 1. Sedentary (reading, working on a computer, watching television, playing cards, etc.) 2. Lightly Active (lawyers, doctors, teachers, laboratory employees, shop workers, many housewives/ husbands and professional musicians) 3. Moderately Active (electricians, carpenters and those in the building trade (excluding heavy laborers), many farm workers, soldiers not in active service, commercial fishermen and housewives/ husbands without mechanical appliances. ) 4. Very active (unskilled laborers, some agricultural workers, forestry, steel and mine workers, solders in active service) 5. Extremely Active/Athletic (lumberjacks, blacksmiths, female construction workers.) |  |
| 115. | Cancer stage | 1. Stage I 3. Stage III 2. Stage II 4. Stage IV |  |
| 116. | Which one of breast affected? | 1. Right 2. Left |  |

**II. Reproductive Factors**

| 201. | At what age did your menstrual periods begin? | 1. Never menstruated 2. 9 or younger 3. 10 4. 11 5. 12 6. 13 7. 14 8. 15 9. 16 10. 17 or older 11. Don’t know |
| --- | --- | --- |
| 202. | Did you use modern contraceptive? | 1. Yes 2. No |
| 203. | Have you ever been pregnant? | 1. Yes 2. No |
| 204. | How old were you when your first child birth? | ____________ Age |
| 205. | Have you ever had stillbirths, miscarriages, abortions? | 1. Yes 2. No |
| 206. | Did you breastfeed any of your children? | 1. Yes 2. No |
| 207. | How long breastfeed your children? | 1. less than6 months  2. up to 6 month  3. up to 1 year  4. up to 2 years and above |
| 208. | How many children do you have? | --------------- |
| 209. | Menopause status | 1. pre-menopause 2. post-menopause |

**III. Food frequency questions**

| **Food Item** | |  | **Frequency** | | | | |
| --- | --- | --- | --- | --- | --- | --- | --- |
|  |  | **Never** | **<once a month** | **1-3/month** | **1-2/week** | **3-4/week** | **Daily** |
| 301. | **Food made of grains:**  Porridge, Oats, Bread, Enjera, Rice, Pasta/Macaroni Or Other Foods Made From Grains, Wheat, Barley, Millet, Sorghum, Maize Kolo |  |  |  |  |  |  |
| 302. | **White roots and tubers and plantains:**  White potatoes, Boye and other foods made from white- fleshed roots or tubers, or plantains, false bananas |  |  |  |  |  |  |
| 303. | **Pulse (beans, peas, lentils, etc.):**  Mature beans or peas( fresh or dried seed),lentils or bean/pea products, including nefro, foods made from bean, lentils, plea, soybean, Chickpea |  |  |  |  |  |  |
| 304. | **Nuts and seeds:**  ground nut/peanut or certain seeds or nut/seed, pumpkinseed, sunflower seed, etc |  |  |  |  |  |  |
| 305. | **Milk and milk products:**  Milk, cheese, yoghurt or other milk products |  |  |  |  |  |  |
| 306. | **Any organs meat:**  Liver, kidney, heart, or other organ meats |  |  |  |  |  |  |
| 307. | **Any other types of meat or poultry:**  Beef, pork, lamb, chicken, |  |  |  |  |  |  |
| 308. | **Any fish whether fresh or dried**  Fresh or dried fish |  |  |  |  |  |  |
| 309. | **Eggs**  Eggs from poultry or any other bird |  |  |  |  |  |  |
| 310. | **Any green leafy vegetables:**  lettuce, spinach, Cabbage, moringa, green pepper etc |  |  |  |  |  |  |
| 311. | **Any vegetables or roots that are orange colored inside like:**  Pumpkin, carrots, sweet potatoes, beets |  |  |  |  |  |  |
| 312. | **Any other vegetables:**  zucchini, tomato, onion, peas, green maize, beans etc |  |  |  |  |  |  |
| 313. | **Any fruits:**  Avocado, papaya, mango, banana, orange, apple, grapes, lemon, mandarin, pineapple etc |  |  |  |  |  |  |
| 314. | **Any oil and fats:**  Oil, fats or butter added food or used for cooking, animal fats |  |  |  |  |  |  |
| 315. | **Any savory and fried snacks**  Chips, fried bread, other fried snack |  |  |  |  |  |  |
| 316. | **Any sweets such as:**  Sugary foods, such as chocolates, candies, cookies/sweet biscuit and cakes, ice cream |  |  |  |  |  |  |
| 317. | **Any sweetened beverages:**  Sweet tea or coffee with sugar, sweetened fruit juice |  |  |  |  |  |  |
| 318. | **Any soft drinks and soda:**  Coca cola, Mirinda, Pepsi |  |  |  |  |  |  |
| 319. | **Any condiments and seasonings such as:**  Ingredients used in small quantities for flavor, such as chilies spices, herbs, seeds |  |  |  |  |  |  |
| 320. | **Any canned and processed foods:**  Canned fruit juices, canned tomato, etc |  |  |  |  |  |  |
| 321. | **Any other alcoholic beverages:**  beer, wine, tela, areke |  |  |  |  |  |  |

**Thank you for your responses!!!**

**Amharic version of the questionnaires**

7. አባሪ

የአማርኛ መጠይቅ

ጤና ይስጥልኝ፡፡ ስሜ ------------------ ይባላል፡፡ እኔ በባህር ዳር ዩኒቨርሲቲ የቴክኖሎጂ ኢንስቲትዩት ከስነ-ምግብ ትምህርት ክፍል የመጣሁ ስሆን ስለ አጠቃላይ ጤንነትዎ እና የአመጋገብ ሁኔታ የተመለከቱ አንዳንድ ጥያቄዎችን እጠይቅዎታለሁ :: በተጨማሪም ቁመትዎን እና ክብደትዎን እለካለሁ። ቃለመጠይቁ 30 ደቂቃዎችን አካባቢ ይወስዳል ፡፡ ለዚህ ሥራ ስኬታማነት እና ለወደፊት ለሚደረጉ ጥናቶችና ፕሮግራሞች የእርስዎ ተሳትፎ ትልቅ ሚና ይኖረዋል ፡፡ በዚህ ቃለ-ምልልስ ውስጥ የእርስዎ ተሳትፎ በፈቃደኝነት ላይ የተመሰረተ ነው ፡፡ መጠይቁ እየተሞላ ማቑረጥ ቢፈለልጉ በማነኛዉም ሰዓት ቃለመጠይቁን የመተው ሙሉ መብት አለዎት ፡፡ ከእርስዎ የምንሰበስበው መረጃ ሙሉ በሙሉ በሚስጥር የሚያዝ ይሆናል፡፡ እኔ የምሰበስበው መረጃ ለዚህ ምርምር ብቻ የሚውል ሲሆን ስምዎት እና ማንነትዎ በሪፖርቱ ውስጥ አይገለጽም ፡፡ በመጨረሻም በጥናቱ ውስጥ በመሳተፍዎ የሚከሰት ምንም ዓይነት አደጋ እንደሌለ ማረጋገጥ እፈልጋለሁ ፡፡

ስለ ዳሰሳ ጥናቱ ማንኛውም ጥያቄ ካለዎት እኔ ለመመለስ ዝግጁ ነኝ ፡፡ ጥያቄዎች አሉዎት? እባክዎን የገለፅኩት ማንኛውም ነገር ግልፅ አለመሆኑን ያሳውቁኝ እና እርስዎ እንዲገነዘቡት የበለጠ ለማብራራት ደስተኛ ነኝ ፡፡

ስለዚህ ልዩ ምርምር ዓላማ እና አጠቃቀም መረጃ ተሰጥቶኛል ፡፡ ከነዚህ ሁሉ በኋላ ተረድቻለሁ እና

1. በዚህ ምርምር በፈቃደኝነት ለመሳተፍ እስማማለሁ

2. በዚህ ምርምር ውስጥ ለመሳተፍ አልስማማም

ቀን _________

ለጥናቱ ላደረጉት ትብብር በቅድሚያ አመሰግናለሁ

ጥያቄ

ካርድ ቁጥር. __________

ቃለ-መጠይቅ አድራጊ ስም __________________________ ቀን ____________

**I. የግል ፣ ማህበራዊ ፣ ሥነ-ህዝብ ፣ አንትሮፖሜትሪክ እና ክሊኒካዊ መረጃ**

| **No** | **ጥያቄዎች** | **ኮድ** | **ምርመራ** |
| --- | --- | --- | --- |
| 101. | ዕድሜ | _______________ |  |
| 102. | ቁመት | _________ሜትር |  |
| 103. | ክብደት | _________ኪግ |  |
| 104. | ሃይማኖትዎ ምንድን ነው | 1. ኦርቶዶክስ 4. አድቬንቲስት  2. ሙስሊም 5. ካቶሊክ  3. ፕሮቴስታንት 6. ሌላ ካለ |  |
| 105. | የአሁኑ የትዳር ሁኔታዎ ምንድን ነው? | 1.ያላገባች 3. የተፋታች  2. ያገባች 4. የሞተባት |  |
| 106. | የመኖሪያ ቦታ? | 1. ከተማ 2. ገጠር 3. ከፊል-ከተማ |  |
| 107. | የትምህርት ደረጃዎ ምንድ ነው? | 1. ማንበብና መጻፍ የማይችሉ  2. ማንብና መፃፍ የሚችሉ  3. የመጀመሪያ ደረጃ (1-4)  4. የመጀመሪያ ደረጃ (5-8)  5. ሁለተኛ ደረጃ (9-12)  6. ኮሌጅ እና ከዚያ በላይ  7. ሌሎች (ይግለጹ) |  |
| 108. | ሥራዎ ምንድ ነው? | 1. የቤት እመቤት 5. የግል ስራ  2. የቀን ሠራተኛ 6. የመንግስት ሰራተኛ  3. ገበሬ 7. ሌላ (ይግለጹ) ________  4. ነጋዴ |  |
| 109. | ወርሃዊ የቤተሰብዎ የገቢ መጠን ምን ያህል ነው? | ______________________ |  |
| 110. | ሲጋራ ያጨሳሉ? | 1. አዎ 2. የለም |  |
| 111. | ከወላጆቻችሁ ፣ ከእህት ወይም ከልጆቻችሁ መካከል የጡት ካንሰር ያለው ነበረ? | 1. አዎ  2. የለም  3. አላውቅም | አላውቅም ከሆነ ወደ ጥያቄ ቁ113 ይሂዱ |
| 112. | አዎ ከሆነ ያለዎት ዝምድና ምንድን ነው? | _______________________ |  |
| 113. | በደረት ወይም በጡት ላይ የጨረር ሕክምና አድርገው ያውቃል? | 1. አዎ  2. የለም |  |
| 114. | የአካል ብቃት እንቅስቃሴዎን ደረጃ እንዴት ይመዘኑታል? | 1. ቁጭ ብሎ (ንባብ ፣ ኮምፒተር ላይ መሥራት ፣ ቴሌቪዥን ማየት ፣ ካርታ መጫወት ፣ ወዘተ)  2. ቀለል ያሉ (ጠበቆች ፣ ሐኪሞች ፣ መምህራን ፣ የላብራቶሪ ሠራተኞች ፣ የሱቅ ሠራተኞች ፣ ብዙ የቤት እመቤቶች / ባሎች እና ሙያዊ ሙዚቀኞች)  3. በመጠነ- ንቁ (ኤሌክትሪክ ሰሪዎች ፣ አናጢዎች እና በግንባታ ንግድ ውስጥ ያሉ (ከባድ የጉልበት ሠራተኞችን ሳይጨምር) ፣ የእርሻ ሠራተኞች ፣ ንቁ አገልግሎት የማይሰጡ ወታደሮች ፣ የንግድ ሥራ እና የቤት እመቤቶች / ባል ያለ ሜካኒካዊ መሳሪያዎች))  4. በጣም ንቁ (ችሎታ የሌላቸው የጉልበት ሠራተኞች ፣ አንዳንድ የግብርና ሠራተኞች ፣ የደን እና የማዕድን ሠራተኞች)  5. እጅግ በጣም ንቁ / አትሌቲክስ (ጣውላ ሰሪዎች ፣ አንጥረኞች ፣ ሴት የግንባታ ሠራተኞች ፡፡) |  |
| 115. | የካንሰር ደረጃ | 1. ደረጃ I 3. ደረጃ III  2. ደረጃ II 4. ደረጃ IV |  |
| 116. | የትኛው ጡትዎ ነው የታመመው? | 1. የቀኝ 2. የግራ |  |

**II. ስነ-ተዋልዶዊ ምክንያቶች**

| 201. | የወር አበባ ማየት በስንት ዓመትዎ ጀመሩ? | 1. የወር አበባ አይቸ አላዉቅም 7. 14  2. 9 ወይም ከዚያ በታች 8. 15  3. 10 9. 16  4. 1110. 17 ወይም ከዚያ በላይ  5. 12 11. አላውቀውም  6. 13 |
| --- | --- | --- |
| 202. | ዘመናዊ የወሊድ መከላከያ ይጠቀሙ ነበር? | 1. አዎ 2. የለም |
| 203. | አርግዘው ያውቃሉ? | 1. አዎ 2. የለም |
| 204. | የመጀመሪያ ልጅዎትን ሲወልዱ ዕድሜዎ ስንት ነበር? | ____________ ዕድሜ |
| 205. | ሞቶ የተወለደ ልጅ፣ ፅንስ ማስወረድ ወይም ፅንስ ማቑረጥ አጋጥሞዎት ያውቃል? | 1. አዎ 2. የለም |
| 206. | ሁሉንም ልጆችዎን ጡት ያጠቡ ነበር? | 1. አዎ 2. የለም |
| 207. | ልጆችዎን ለምን ያህል ጊዜ ነው ጡት ያጠቡ? | 1. ከ 6 ወር በታች  2. እስከ 6 ወር 3. እስከ 1 ዓመት  4. እስከ 2 ዓመት እና ከዚያ በላይ |
| 208. | ስንት ልጆች አሏችሁ | _______ |
| 209. | የማረጥ ሁኔታ | 1. ቅድመ-ማረጥ 2. ድህረ-ማረጥ |

**III. የምግብ አመጋገብ ድግግሞሽ ጥያቄዎች**

| **የምግብ አይነት** | | **ድግግሞሽ** | | | | | |
| --- | --- | --- | --- | --- | --- | --- | --- |
|  |  | **በጭራሽ** | **በወር ከ<1 ጊዜ** | **1-3/**  **በወር** | **1-2/**  **ሳምንት** | **3-4/**  **ሳምንት** | **በየቀኑ** |
| 301. | ከእህል የተሰራ ምግብ  ገንፎ ፣ አጃ ፣ ዳቦ ፣ እንጀራ ፣ ሩዝ ፣ ፓስታ / ማካሮኒ ወይንም ሌሎች ከጥራጥሬ ፣ ስንዴ ፣ ገብስ ፣ ዳጉሳ ፣ ማሽላ ፣ ከበቆሎ ቆሎ የተሰሩ ምግቦች |  |  |  |  |  |  |
| 302. | ነጭ ሥሮች እና ሀረጎች  ነጭ ድንች ፣ ቦዬ እና ሌሎች ከነጭ ሥራ ሥሮች ወይም ከሐሰተኛ ሙዝ የተሠሩ ነጭ ምግቦች |  |  |  |  |  |  |
| 303. | ጥራጥሬዎች (ባቄላ ፣ አተር ፣ ምስር ፣ ወዘተ)  ንፍሮን ጨምሮ የበሰለ ባቄላ ወይም አተር (ትኩስ ወይም የደረቀ ዘር) ፣ ምስር ወይም የባቄላ / አተር ምርቶች ፣ ከባቄላ የተሰራ ምግብ ፣ አኩሪ አተር |  |  |  |  |  |  |
| 304. | ለውዝ እና ዘሮች  ኦቾሎኒ፣ የሰሊጥ ዘር ፣ የዱባ ዘር ፣ የሱፍ አበባ ዘር ፣ ወዘተ |  |  |  |  |  |  |
| 305. | ወተት እና የወተት ተዋጽኦዎች  ወተት ፣ አይብ ፣ እርጎ ወይም ሌሎች የወተት ተዋጽኦዎች |  |  |  |  |  |  |
| 306. | ማንኛውም የአካል ክፍሎች ሥጋ  ጉበት ፣ ኩላሊት ፣ ልብ ፣ ወይም ሌላ የአካል ሥጋ |  |  |  |  |  |  |
| 307. | ሌላ ማንኛውም ስጋ  የበሬ ሥጋ ፣ የአሳማ ሥጋ ፣ የበግ ሥጋ ፣ የዶሮ ስጋ |  |  |  |  |  |  |
| 308. | ትኩስ ወይም የደረቀ ማንኛውም ዓሳ |  |  |  |  |  |  |
| 309. | እንቁላል  እንቁላል ከዶሮ እርባታ ወይም ከሌላ ከማንኛውም ወፍ |  |  |  |  |  |  |
| 310. | ማንኛውም አረንጓዴ ቅጠል ያላቸው አትክልቶች  ሰላጣ ፣ቆስጣ፣ ጎመን ፣ ሞሪንጋ ፣ ቃሪያ ወዘተ |  |  |  |  |  |  |
| 311. | በውስጣቸው ብርቱካናማ ወይም ቢጫ ቀለም ያላቸው ማናቸውም አትክልቶች ወይም ሥሮች  ዱባ ፣ ካሮት ፣ ስኳር ድንች ፣ ቀይ ስር |  |  |  |  |  |  |
| 312. | ሌሎች አትክልቶች  ዝኩኒ ፣ ቲማቲም ፣ ሽንኩርት ፣ የባቄላ የአተር (የፎሶሊያ) ፣እና የበቆሎ እሸቶች ወዘተ |  |  |  |  |  |  |
| 313. | ፍራፍሬዎች  አቮካዶ ፣ ፓፓያ፣ ማንጎ፣ ሙዝ ፣ ብርቱካን ፣ አፕል ፣ ወይን ፣ ሎሚ ፣ ማንዳሪን ፣ አናናስ ወዘተ |  |  |  |  |  |  |
| 314. | ማንኛውም ዘይት እና ቅባቶች  ዘይት ፣ ቅባት ወይም ቅቤ የተጨመረበት ምግብ ወይም ለምግብ ማብሰያ የእንስሳት ስብ |  |  |  |  |  |  |
| 315. | ማንኛውም ጣፋጭ እና የተጠበሱ ምግቦች  ቺፕስ ፣ የተጠበሰ ዳቦ ፣ ሌላ የተጠበሰ መክሰስ |  |  |  |  |  |  |
| 316. | ማንኛውም ጣፋጮች  ቸኮሌቶች ፣ ከረሜላዎች ፣ ኩኪሶች / ጣፋጭ ብስኩት እና ኬኮች ፣ ወይም አይስክሬም የመሳሰሉ የስኳር ምግቦች |  |  |  |  |  |  |
| 317. | ማንኛውም የስኳር ጣዕም ያላቸው መጠጦች  ጣፋጭ ሻይ ወይም ቡና በስኳር ፣ ጣፋጭ የፍራፍሬ ጭማቂ |  |  |  |  |  |  |
| 318. | ማንኛውም ለስላሳ መጠጦች እና ሶዳ  ኮካ ኮላ ፣ ሚሪንዳ ፣ ፔፕሲ ወዘተ |  |  |  |  |  |  |
| 319. | ማንኛውም ቅመማ ቅመሞች  የዕፅዋት ዘሮች የመሳሰሉት ለጣዕም በትንሽ መጠን የሚያገለገሉ ንጥረ ነገሮች |  |  |  |  |  |  |
| 320. | ማንኛውም የታሸጉ እና የተሰሩ ምግቦች  የታሸጉ የፍራፍሬ ጭማቂዎች ፣ የታሸገ ቲማቲም ፣ ወዘተ |  |  |  |  |  |  |
| 321. | ሌላ ማንኛውም የአልኮል መጠጦች  ቢራ ፣ ወይን ፣ ጠላ ፣ አረቄ |  |  |  |  |  |  |

**ለምላሽዎ አመሰግናለሁ!!!**
